# Real-time spectral evaluation for gas chromatography–Fourier transform infrared spectrometry

**DOI:** 10.1155/S1463924687000154

**Published:** 1987

**Authors:** Robert L. White

**Affiliations:** Department of Chemistry University of Oklahoma Norman Oklahoma 73019 USA


**Journal of Automatic Chemistry**/*Journal of Clinical Laboratory Automation*, **Vol. 9, No. 2 (April–June 1987), pp. 66–71**

### Real-time spectral evaluation for gas chromatography—Fourier transform infra-red spectrometry

**Robert L. White**

*Department of Chemistry, University of Oklahoma, Norman, Oklahoma 73019, USA*

### Introduction

Infra-red analysis of gas chromatographic eluents (GC/FT-IR) can provide tentative structure identification in addition to component detection [1–6]. Large quantities of useful information are generated during GC/FT-IR analysis. Typical GC/FT-IR analyses produce thousands of interferograms. Days of scrutiny may be required before all useful information contained in this data is extracted and interpreted. The potential of GC/FT-IR for routine complex mixture analysis has motivated researchers to investigate possible methods for reducing operator intervention in the data evaluation process [7–10]. Recent emphasis has been placed on developing sensitive algorithms for generating gas chromatograms from interferometric information [11–16]. Previous automated component detection and identification systems were limited to off-line applications because data collection requirements were incompatible with data evaluation operations [7].

The GC/FT-IR data collection/evaluation system described here features simultaneous interferogram acquisition and spectral interpretation. The data system incorporates dual microprocessors with multitasking and parallel computing capabilities. One of the processors is dedicated to signal averaging interferogram data points. The other microprocessor is used for on-line gas chromatogram generation, interferogram data file storage, and data reduction. Data system software is organized for maximum flexibility. Data reduction procedures can be tailored for a specific analysis by modifying a general-purpose macro program. Concurrently, active processes are arbitrated by priority assignment. The highest priority is given to on-line gas chromatogram generation and interferogram data file storage. Lower priority is given to data reduction.

### Materials and methods

### *Apparatus*

A Hewlett-Packard 5890 gas chromatograph equipped with a split/splitless capillary column injector was used for mixture separations. Alcohol mixtures were separated using a 25 m × 0·31 mm Hewlett-Packard Ultra-1 capillary column with a 0·52 μm methyl silicone stationary phase film thickness. Column flow rate was adjusted to 2 ml/min and the chromatographic oven was temperature programmed from 40 °C to 150 °C at a rate of 20 °C/min after an initial isothermal period at 40 °C for 1 min. The infra-red spectrometer used for GC/FT-IR measurements was a Mattson Instruments, Inc. Sirius 100 FT-IR. The FT-IR was equipped with a narrow band mercury–cadmium–telluride (MCT) detector and was operated at 8 $cm^{-1}$ resolution (2048 data points per interferogram) using an interferometer mirror scan velocity of 2·4 cm/s. Software written in C programming language [17 and 18] was developed for FT-IR data collection and spectral evaluation. Macro programs were written using UNIX C-Shell format (19).

The GC/FT-IR interface constructed by the author's laboratory is described in detail elsewhere [20]. The interface consists of a gold-coated light pipe contained inside a rectangular aluminum oven. The light pipe interface is connected to the gas chromatographic column by using heated nickel transfer tubing (figure 1). Potassium bromide windows are attached to the ends of the light pipe using high-temperature epoxy cement.

### *Data acquisition*

Hardware used for GC/FT-IR data collection is shown schematically in figure 2. Interferogram information was sampled at a rate of 80 kHz using a 16 bit ADC. This data was transferred to CPU 2 via a parallel communication interface. Signal averaging was accomplished using the dual ported, double buffered memory labelled MEM A and MEM B in figure 2. Program execution requests were handled by CPU 1 which employed a UNIX operating system. CPU 1 performed chromatogram generation, interferogram data file storage, and data reduction functions. A special memory location was designated as a communication register and was used to transfer messages between the two microprocessors and arbitrate control of the dual ported, double buffered memory.

Table 1 summarizes a typical GC/FT-IR data collection sequence. Prior to the start of a GC/FT-IR separation, signal averaging software was transferred from CPU 1 to CPU 2. This was accomplished by using the dual ported buffer memory (MEM A). Signal averaging object code was placed into MEM A by CPU 1. CPU 1 then communicated with CPU 2 via the communication register and instructed CPU 2 to initiate the program contained in MEM A. The first operations that the program performed were to copy itself to a memory buffer, which was dedicated to CPU 2 (MEM 2), and reset the program counter to a memory location in that buffer. A timer was initialized at the start of data acquisition. This timer was read each time an interferogram was collected and the retention time (milliseconds)

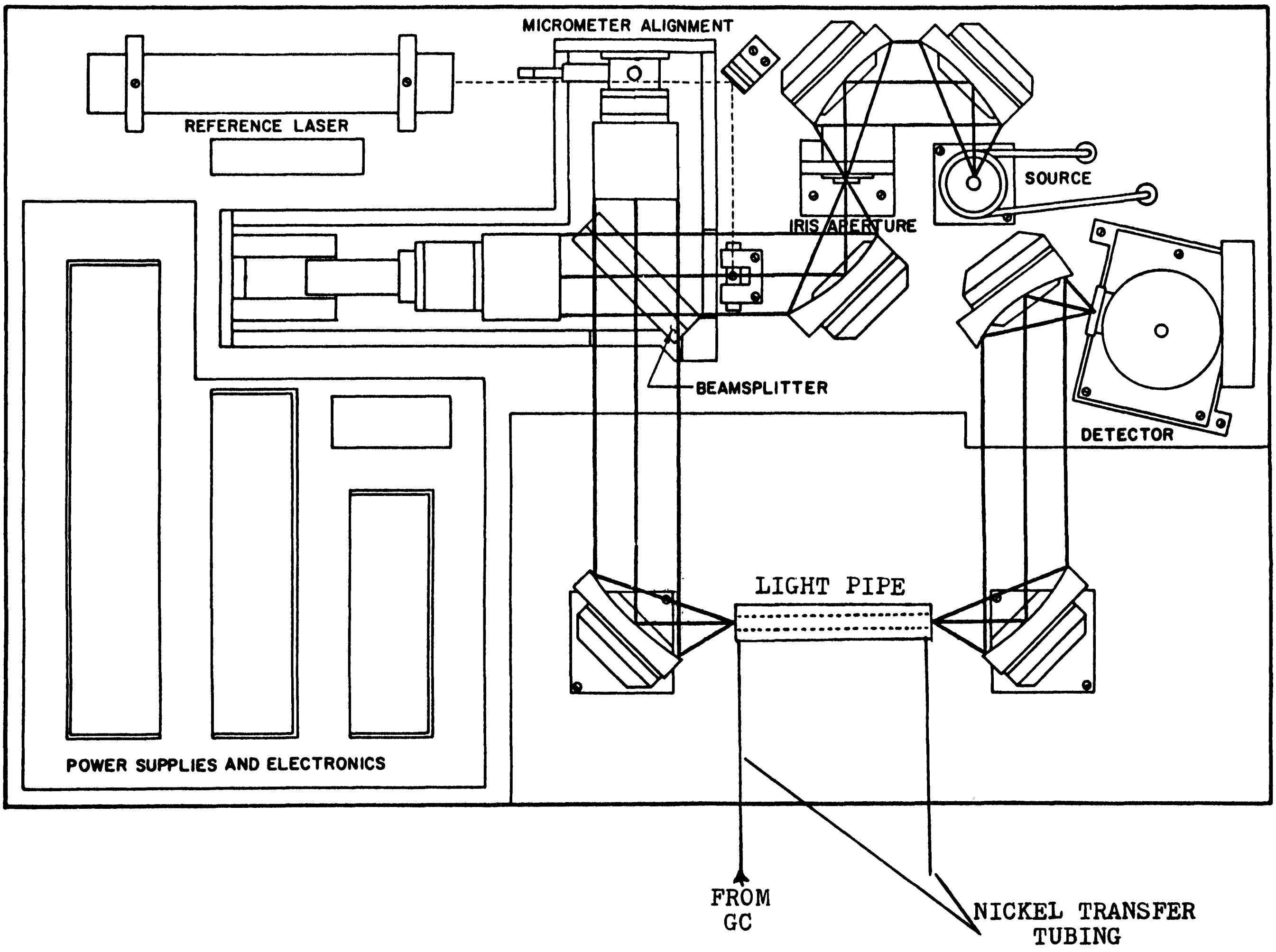

*Figure 1. Diagram of the GC/FT-IR light pipe interface.*

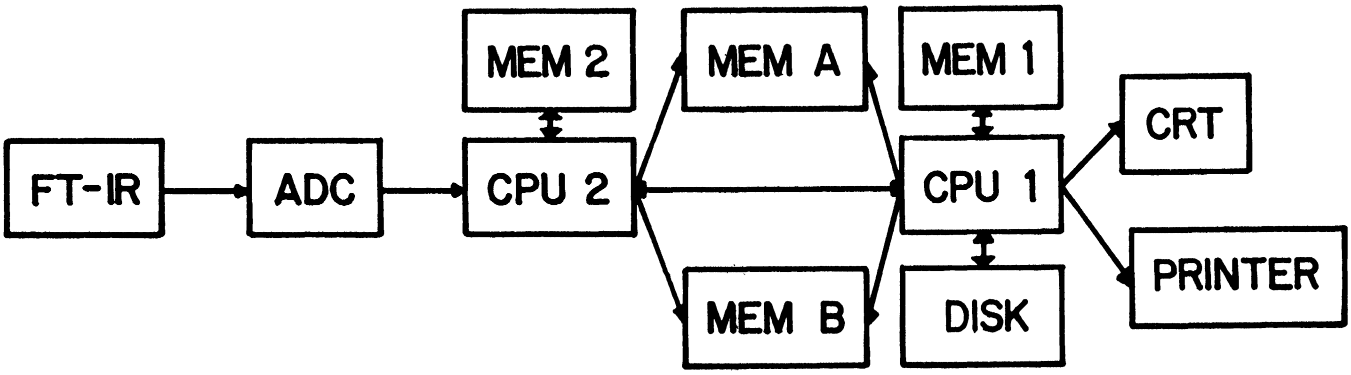

*Figure 2. Block diagram of the GC/FT-IR data collection/evaluation system.*

for that data was placed in memory immediately following the interferogram data. The signal averaging program collected data points generated by the 16 bit ADC and added this data to previously collected data stored in MEM A. When the required number of scans had been signal averaged, CPU 2 informed CPU 1 that a completed data set was available in MEM A. CPU 1 then acquired control of MEM A and relinquished control of MEM B to CPU 2. CPU 2 continued signal averaging and stored information in MEM B. During the signal averaging period, CPU 1 used the previously collected data (MEM A) to compute a chromatogram intensity value, check chromatogram data for possible eluting components, and store the interferogram data on magnetic disk storage media. Typically, CPU 1 finished its tasks before CPU 2 had completed signal averaging. CPU 1 then waited for a message from CPU 2 indicating that signal averaging was complete. When this occurred, another chromatogram generation and data storage cycle was performed. Maximum data acquisition efficiency was

*Table 1. Typical microprocessor conversation and parallel processing.*

| Function | CPU 1 | CPU 2 |
|---|---|---|
| 1 | Load data collection software in MEM A | — |
| 2 | Relinquish MEM A | — |
| 3 | — | Accept MEM A |
| 4 | Wait for CPU 2 to finish | Signal average |
| 5 | — | Relinquish MEM A |
| 6 | Accept MEM A | — |
| 7 | Relinquish MEM B | — |
| 8 | Chromatogram generation | Accept MEM B |
| 9 | Data file storage | Signal average |
| 10 | Wait for CPU 2 to finish | Relinquish MEM B |
| 11 | Accept MEM B | — |
| 12 | Relinquish MEM A | — |
| 13 | Chromatogram generation | Accept MEM A |
| 14 | Data file storage | Signal average |
| 15 | Wait for CPU 2 to finish | ,, |
| 16 | ,, | ,, |
| 17 | ,, | ,, |
| 18 | ,, | ,, |
| 19 | ,, | ,, |

attained when the time required for signal averaging was greater than the time required for CPU 1 to complete its tasks because CPU 2 did not have to wait for CPU 1 before resuming data collection.

Four data files were stored during data acquisition. Two files were created prior to the beginning of the separation. One of these files contained FT-IR scan conditions used to collect subsequent interferograms. The other file contained the five background interferograms used to compute Gram-Schmidt basis vectors. The other two files were opened at the start of the separation and filled with information during GC/FT-IR data acquisition. One of the files contained Gram-Schmidt chromatogram intensity values and retention times, the other contained interferogram data. After separation, the chromatogram was displayed on the CRT and a cursor was used to select interferogram data for viewing and additional processing.

### Chromatogram generation

Gram-Schmidt vector orthogonalization was used to generate gas chromatograms from interferometric data during separations. The Gram-Schmdit orthogonalization technique does not require Fourier transformation of interferogram data prior to chromatogram intensity calculation [11–16]. The technique has been shown to be more sensitive than integrated absorbance methods and requires significantly fewer mathematical operations than Fourier transformation [15]. Prior to starting GC/FT-IR data collection, five interferograms were collected to characterize gas chromatographic background. These interferograms were used to form basis vectors for subsequent Gram-Schmidt orthogonalizations. Vectors were extracted from interferograms starting 35 data points past the zero retardation point and extending 25 data points. Using these vector designations, Gram-Schmidt orthogonalizations required 275 floating point multiplications and 275 floating point additions for each computed chromatogram intensity. In contrast, a 32 $cm^{-1}$ spectrum computed by Fourier transformation would require 3700 multiplications and 9300 additions [15]. Thus, employing Gram-Schmidt orthogonalization for real-time chromatogram generation significantly reduced processing time required for intensity computation. As a result, sufficient time was available for data reduction operations during GC/FT-IR data acquisition.

### Chromatographic peak detection

Gas chromatographic peaks were detected in real-time using a point-by-point slope comparison method. A flow chart for the peak detection algorithm is depicted in figure 3. Each chromatogram intensity (CURRENT) was compared with the previous measured value (LAST) and the slope of the chromatogram was calculated. Previous slope tendencies were saved for comparison by setting software flags (POS_SLOPE, NEG_SLOPE) to true or false as appropriate. A positive slope indicated the beginning of a possible chromatographic peak elution. When a positive slope was first encountered, the chromatogram intensity at this point was saved (START) and subsequent intensity values were compared until a negative slope was computed. When this occurred, the maximum chromatogram intensity of the elution was saved (PEAK) and the next occurrence of a positive slope was sought. The next positive slope marked the end of the potential chromatographic peak elution. Discrimination between chromatogram base-line noise fluctuations and valid chromatographic peaks was based on comparisons of peak height with a pre-set threshold (THRESH). If both the leading (P_THRESH) and trailing (N_THRESH) peak heights exceeded the threshold, the chromatogram peak was assumed to be real. Interferograms collected at the beginning of peak elution

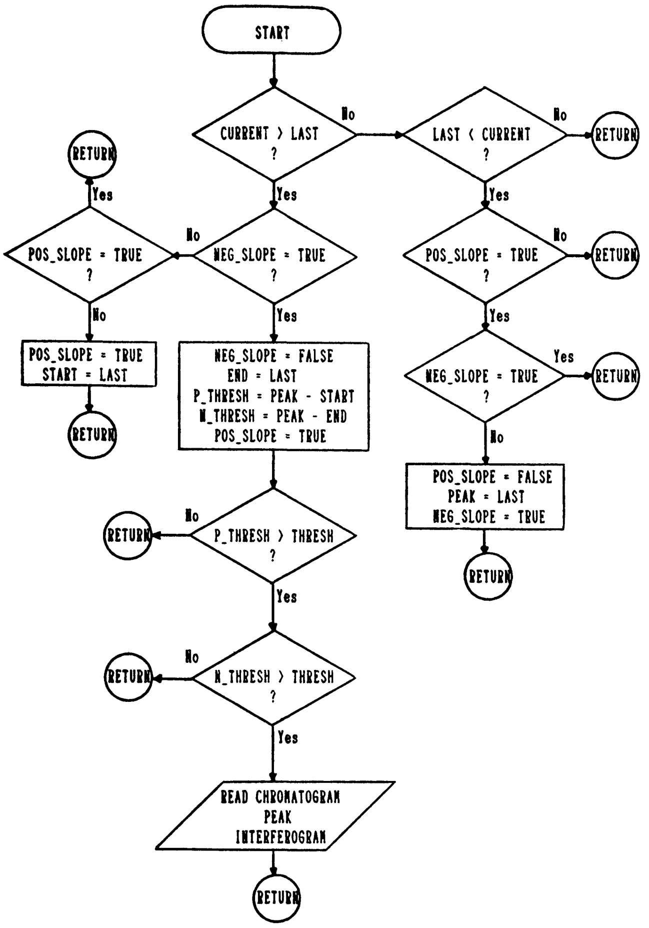

*Figure 3. Flow chart of the algorithm used to detect mixture component elutions during GC/FT-IR data acquisition.*

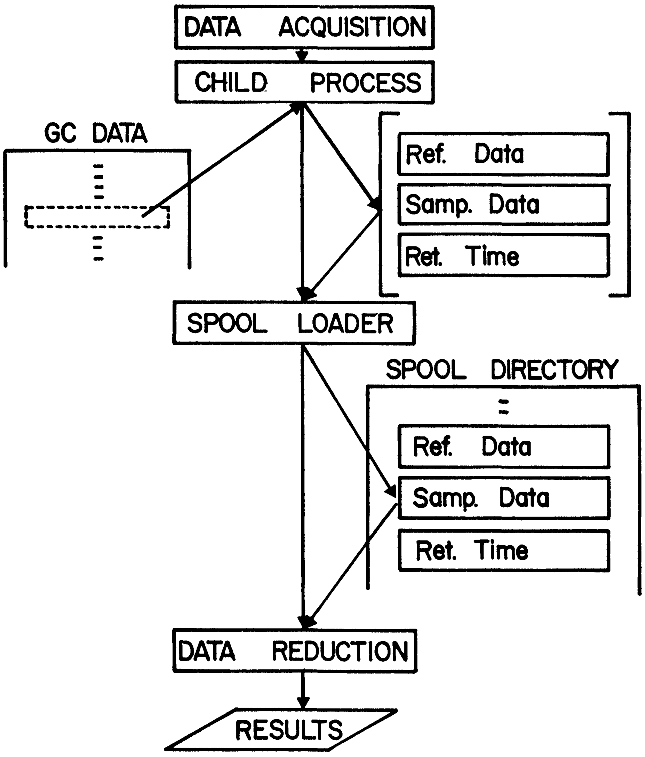

*Figure 4. Interferogram processing sequence for detected mixture components.*

(START) and at the peak maximum (PEAK) were retrieved from disk and submitted for data reduction as reference and sample interferograms respectively. Reference interferograms were selected in this manner to minimize the effect of FT-IR instability during the gas chromatographic separation which could produce base-line artifacts in infra-red spectra computed with reference interferograms acquired prior to the start of gas chromatographic separation. The slope comparison peak detection algorithm required minimal calculation and was insensitive to chromatogram base-line fluctuations.

*Interferogram data reduction*

The high data acquisition rate required for GC/FT-IR measurements made it impossible to perform intensive data reduction operations with the data collection program. Instead, independent 'child' processes were created by the data collection program when data reduction was required. More than one child process could be active at one time. Additional processes to load the data reduction spool directory with appropriate interferogram data were initiated by 'child' processes. All of these processes operated at lower priority than the data collection program. Data reduction was interrupted whenever the processor was needed for data acquisition functions. Figure 4 depicts the data processing sequence initiated when chromatographic peaks were detected.

Child processes were formed by the parent data collection program by making a copy of itself and executing the copy program as a low priority background process. Since child processes were exact copies of the parent, each child process had access to all disk files opened by the parent. Therefore, child processes had direct access to interferogram data previously collected during the GC/FT-IR separation. Prior to child process initiation, the child process program counter was placed at the beginning of a subroutine which retrieved reference and sample interferogram data files from the chromatogram disk file and stored this data along with retention time as separate files. When data transfer was completed, the child process invoked the spool directory loading program and then automatically aborted. The spool directory loading program copied the extracted interferograms to the data reduction spool directory (figure 5). When file copying was completed, the loader program checked if the data reduction program was active. If it was inactive, the loader program initiated it. The data reduction macro program removed appropriate interferogram data files from the spool directory and processed this data according to the procedures specified (figure 6). Data reduction procedures could be altered by simply changing the programs referenced in the data reduction macro program. Since interferogram data placed in the spool directory was in the same form as normal FT-IR data files, existing FT-IR software could be used for data reduction applications. In fact, Fourier transformation of interferogram data to produce infra-red spectra was accomplished using the 'process' program supplied with the FT-IR with no modification.

```
#
### spool_fil - fill spool directory with interferogram data
#

J="/usr/spool/search/"     # location of the spool directory
for file in $*
do
     mv $file.big /usr/spool/search/$file.big     # ref. data
     mv $file.igm /usr/spool/search/$file.igm     # samp. data
     mv $file.ret /usr/spool/search/$file.ret     # ret. time
done

### check if data reduction macro is executing if test ! -f /usr/spool/search/lock then

     echo -n "lock" > /usr/spool/search/lock

     /usr/bin/reduce &     # start data reduction fi

```

*Figure 5. Spool loader macro program.*

```
#
### reduce - data reduction daemon
#

J="/usr/spool/search/*.igm"     # samp. data

K="/usr/spool/search/*.big"     # ref. data

L="/usr/spool/search/*.ret"     # ret. time

### perform data reduction for all data in spool directory while test "`eval echo $J`" != '/usr/spool/search/*.igm'

do     # move data files to another directory for processing

      for file in $J

      do

            mv $file /usr/spool/search/spectrum/spc.igm

            break

      done

      for file in $L

      do

            mv $file /usr/spool/search/spectrum/spc.ret

            break

      done

      for file in $K

      do

            mv $file /usr/spool/search/spectrum/spc.big

            rm /usr/spool/search/spectrum/spc.bkg

                  # perform data processing

                      # Fourier Transformation

                  /usr/bin/process /usr/spool/search/spectrum/spc

                      # Library Search

                  /usr/bin/search /usr/spool/search/spectrum/spc.abs

            break

      done

      J="/usr/spool/search/*.igm"

done

### when all files are removed from spool directory, abort rm -f /usr/spool/search/lock
```

*Figure 6. Data reduction macro program.*

### Library search

For qualitative analysis, infra-red spectra obtained from GC/FT-IR analysis were identified using library search comparisons with the 3300 spectra EPA vapor phase library. The search program was written in C language and was designed to be used with FT-IR spectra generated in the normal operation of the instrument. All library spectra were 16 $cm^{-1}$ resolution and only information over the range 4000 $cm^{-1}$ to 700 $cm^{-1}$ was saved. GC/FT-IR spectra were compared with library spectra by computing the sum of the squares of differences between GC/FT-IR and library spectra (square difference metric [10]). Search results were ordered in increasing square difference. The top 10 search matches were printed for each search. Searches could be confined to specified wavelength regions to reduce search times.

*Table 2. Alcohol real-time search results.*

| Peak | Alcohol | Top search match | Alcohol match Position |
|---|---|---|---|
| 1 | Methanol | Methanol | 1 |
| 2 | Ethanol | Ethanol | 1 |
| 3 | t-Butanol | t-Butanol | 1 |
| 4 | 1-Propanol | 1-Propanol | 1 |
| 5 | iso-Butanol | iso-Butanol | 1 |
| 6 | 1-Butanol | 1-Butanol | 1 |
| 7 | 1-Pentanol | 1-Butanol | 10 |
| 8 | 1-Hexanol | 1-Hexanol | 1 |
| 9 | 2-Octanol | 2-Heptanol | 6 |
| 10 | 1-Octanol | 1-Nonanol | 2 |

### Results and discussion

Automatic qualitative analysis capabilities of the GC/FT-IR data system were evaluated by analysing an equal volume mixture of 10 alcohols. A split injection was made into the gas chromatograph which transferred approximately 375 ng of each alcohol to the gas chromatographic column. The first alcohol (methanol) was detected 1·5 min after injection and the entire separation required 9 min. Library searches were performed for the 1800 $cm^{-1}$ to 1000 $cm^{-1}$ spectral range and required 2·4 min during data acquisition. Search results for the first three mixture components were available prior to the end of the separation. After separation was completed, the other seven components were identified by library searches requiring 1 min for each search. The discrepancy in search time periods was caused by data reduction receiving low priority during data acquisition and high priority when data acquisition was finished. Search results for the 10 alcohols is compiled in table 2. Seven of 10 alcohols were correctly identified by the top library search match. Homologues differing by one carbon atom were best matches for the other three alcohols. Search results for 1-pentanol were particularly poor. 1-Pentanol was listed as the tenth library match. Search results for the full spectral range (4000 $cm^{-1}$ to 700 $cm^{-1}$) did not include 1-pentanol in the top 10 matches. Comparison of the measured 1-pentanol spectrum with the 1-pentanol library spectrum indicated significant differences which were responsible for the poor match. Obviously, success of automatic qualitative analysis is highly dependent on the quality of the library used for searching.

In order to test the quantitative analysis capability of the GC/FT-IR software system and demonstrate its flexibility, a program was written to provide quantitative analysis of methanol and ethanol based on infra-red absorbance measurements. Percentage composition was determined by comparing absorbance of the 1058 $cm^{-1}$ band in alcohol spectra with previously generated calibration curves. Calibration curves were made by plotting GC/FT-IR peak absorbance at 1058 $cm^{-1}$ against percentage concentration over a range of 1–10%. For methanol this resulted in a straight line (correlation coefficient = 0·997) with a slope of 0·0518 A/% and a $y$-intercept of −0·002 A. The ethanol calibration curve was also linear (correlation coefficient = 0·998) with a slope of 0·0274 A/% and a $y$-intercept of −0·006 A. Since calibration curve slopes were unequal, it was necessary to distinguish methanol from ethanol prior to concentration calculation. This was accomplished by specifying a retention time window for each component. Time windows were specified for which each of the alcohols were known to elute using the separation conditions. The quantitative analysis program checked the peak retention time prior to data reduction. Interferogram Fourier transformation and concentration calculations were performed only for GC/FT-IR peak spectra which met retention time requirements. All other interferograms were not processed. Quantitative analysis results for selected alcohol mixtures are contained in table 3. Each of the mixtures contained varying amounts of alcohols listed in table 2. Retention time windows were 98s–102s for methanol and 105s–110s for ethanol. In each case, percentage composition for methanol and ethanol were printed before all 10 alcohols were separated. Concentration values were typically obtained within 1 min after each peak had eluted.

### Conclusion

When parallel processing is incorporated into GC/FT-IR data acquisition, there is sufficient time for the least used processor to perform data reduction operations. Using multi-tasking and assignment of priorities, data reduction can be interrupted automatically by the operating system when data acquisition functions are required. The GC/FT-IR data acquisition/evaluation system described here makes efficient use of both microprocessors and produces useful spectral interpretations before gas chromatographic separation is complete. As a result, the number of repetitive tasks that the operator must perform is greatly reduced and useful information is generated rapidly. For gas chromatographic separations requiring long periods of time, the described system provides qualitative or quantitative analysis of components eluting at the beginning of the separation before the separation is complete. The operator need not wait until the separation is completed before interpreting data.

*Table 3. Quantitative analysis of methanol and ethanol*

| Sample No. | % Methanol (actual) | % Methanol (measured) | % Ethanol (actual) | % Ethanol (measured) |
|---|---|---|---|---|
| 1 | 1·00 | 0·72 | 2·00 | 2·16 |
| 2 | 2·00 | 2·01 | 3·00 | 3·12 |
| 3 | 3·00 | 3·05 | 4·00 | 4·07 |
| 4 | 4·00 | 4·06 | 5·00 | 5·13 |

The modular organization of the software facilitates easy configuration for highly specific data reduction procedures. Advantages of the described system are derived from isolation of data reduction from data collection. Data reduction is not subject to the time constraints of data acquisition. This permits utilization of virtually any data reduction algorithm desired by the operator, regardless of the number of computations required.

### Acknowledgement

The author wishes to thank Mattson Instruments, Inc. for providing the vapor phase library.

### References

1. Gurka, D. F., *Applied Spectroscopy*, **39** (1985), 827.
2. Gurka, D. F., Umana, M., Pellizzari, E. D., Moseley, A. and de Haseth, J. A., *Applied Spectroscopy*, **39** (1985), 297.
3. Cooper, J. R. and Taylor, L. T., *Applied Spectroscopy*, **38** (1984), 366.
4. Gurka, D. F., Hiatt, M. and Titus, R., *Analytical Chemistry*, **56** (1984), 1102.
5. Gurka, D. F., Laska, P. R. and Titus, R., *Journal of Chromatographic Science*, **20** (1982), 145.
6. Gurka, D. F. and Betowski, L. D., *Analytical Chemistry*, **54** (1982), 1820.
7. Hanna A., Marshall, J. C. and Isenhour, T. L., *Journal of Chromatographic Science*, **17** (1979), 434.
8. Cooper, J. R. and Taylor, L. T., *Analytical Chemistry*, **56** (1984), 1989.
9. Sparks, D. T., Lam, R. B. and Isenhour, T. L., *Analytical Chemistry*, **54** (1982), 1922.
10. Lowry, S. R. and Huppler, D. A., *Analytical Chemistry*, **53** (1981), 889.
11. Brissey, G. M., Henry, D. E., Giss, G. N., Yang, P. W., Griffiths, P. R. and Wilkins, C. L., *Analytical Chemistry*, **56** (1984), 2002.
12. White, R. L., Giss, G. N., Brissey, G. M. and Wilkins, C. L., *Analytical Chemistry*, **55** (1983), 998.
13. Owens, P. M., Lam, R. B. and Isenhour, T. L., *Analytical Chemistry*, **54** (1982), 2344.
14. White, R. L., Giss, G. N., Brissey, G. M. and Wilkins, C. L., *Analytical Chemistry*, **53** (1981), 1778.
15. Hanna, D. A., Hangac, G., Hohne, B. A., Small, G. W., Wieboldt, R. C. and Isenhour, T. L., *Journal of Chromatographic Science*, **17** (1979), 423.
16. de Haseth, J. A. and Isenhour, T. L., *Analytical Chemistry*, **49** (1977), 1977.
17. Kernighan, B. W. and Ritchie, D. M., in *The C Programming Language* (Prentice-Hall, Inc., Englewood Cliffs, New Jersey, 1978).
18. Hancock, L. and Krieger, M., in *The C Primer* (McGraw-Hill Book Co., New York, New York, 1982).
19. McGilton, H. and Morgan, R., in *Introducing the UNIX System* (McGraw-Hill Book Co., New York, 1983).
20. Fields, R. E., III and White, R. L., *Applied Spectroscopy* (submitted).

